# Comparison of intra-articular administration of adenosine, lidocaine and magnesium solution and tranexamic acid for alleviating postoperative inflammation and joint fibrosis in an experimental model of knee arthroplasty

**DOI:** 10.1186/s13018-021-02871-y

**Published:** 2021-12-20

**Authors:** Jodie L. Morris, Hayley L. Letson, Peter McEwen, Erik Biros, Constantin Dlaska, Kaushik Hazratwala, Matthew Wilkinson, Geoffrey P. Dobson

**Affiliations:** 1 Orthopaedic Research Institute of Queensland, Townsville, QLD Australia; 2 Heart and Trauma Research Laboratory, Division of Tropical Health and Medicine, College of Medicine and Dentistry,, James Cook University, Townsville, QLD 4811 Australia

**Keywords:** Total knee arthroplasty, Stiffness, Arthrofibrosis, Inflammation, Tranexamic acid

## Abstract

**Background:**

Dysregulated inflammatory responses are implicated in the pathogenesis of joint stiffness and arthrofibrosis following total knee arthroplasty (TKA). The purpose of this study was to compare the effects of intra-articular (IA) administration of tranexamic acid (TXA), an anti-fibrinolytic commonly used in TKA, and ALM chondroprotective solution on postoperative inflammation and joint tissue healing in a rat model of knee implant surgery.

**Methods:**

Male Sprague–Dawley rats (*n* = 24) were randomly divided into TXA or ALM treatment groups. The right knee of each rat was implanted with titanium (femur) and polyethylene (tibia) implants. An IA bolus (0.1 ml) of TXA or ALM was administered after implantation and capsule closure, and before skin closure. Postoperative coagulopathy, haematology and systemic inflammatory changes were assessed. Inflammatory and fibrotic markers were assessed in joint tissue, 28 days after surgery.

**Results:**

Haemostasis was comparable in animals treated with TXA or ALM after knee implant surgery. In contrast to ALM-treated animals, systemic inflammatory markers remained elevated at day 5 (IL-6, IL-12, IL-10, platelet count) and day 28 (IL-1β, IL-10) following surgery in TXA-treated animals. At day 28 following surgery, the extension range of motion of operated knees was 1.7-fold higher for ALM-treated animals compared to the TXA group. Key inflammatory mediators (NF-κB, IL-12, IL-2), immune cell infiltration (CD68^+^ cells) and markers of fibrosis (α-SMA, TGF-β) were also lower in capsular tissue of ALM-treated knees at day 28.

**Conclusion:**

Data suggest that IA administration of ALM is superior to TXA for reducing postoperative systemic and joint inflammation and promoting restoration of healthy joint tissue architecture in a rat model of TKA. Further studies are warranted to assess the clinical translational potential of ALM IA solution to improve patient outcomes following arthroplasty.

**Supplementary Information:**

The online version contains supplementary material available at 10.1186/s13018-021-02871-y.

## Background

Globally, the demand for total knee arthroplasty (TKA) continues to rise, with a projected increase of 182% over the next decade [1]. Acquired idiopathic knee stiffness and arthrofibrosis are debilitating postoperative complications and leading causes for hospital readmission following TKA [2]. Accumulation of fibrotic tissue within the operated knee leads to restricted joint function and pain, with significant impact to patient quality of life [2, 3]. Dysregulation of early inflammatory and immune responses triggered during surgery, and in the first postoperative week, are implicated in the pathogenesis of knee stiffness and arthrofibrosis [4, 5]. Frontline strategies to minimise surgery-induced inflammation and improve joint tissue healing and patient outcomes following TKA are lacking.

Administration of the anti-fibrinolytic tranexamic acid (TXA) is popular in orthopaedic procedures such as TKA and anterior cruciate ligament reconstruction for the purpose of enhancing haemostasis, improving visualisation during surgery and minimising transfusion requirements [6–8]. In addition, due to immunomodulatory effects, TXA reportedly improves patient-reported knee outcomes after surgery and reduces the risk for postoperative complications such as infection and arthrofibrosis [8–11]. TXA can be administered orally, intravenously or topically; the latter route is often cited as a preferred method due to lack of contraindications [7, 12, 13]. However, the immunomodulatory effects of TXA following TKA remain controversial due to conflicting reports of both anti- and pro-inflammatory responses that are likely associated with a lack of standardisation in dosing, scheduling and administration route [7, 12, 14–17]. Further, controversial evidence of toxicity towards chondrocytes and periarticular tissues when TXA is delivered topically have also precluded its routine adoption in TKA [17–19].

ALM solution, a combination of adenosine, lidocaine and Mg^2+^, is an emerging small-volume therapy with anti-ischaemic, anti-inflammatory, coagulopathy corrective, chondroprotective and anti-fibrotic properties [20–27]. Recently, intra-articular (IA) administration of ALM was shown to modulate inflammatory responses in the early postoperative period and alleviate post-TKA fibrosis in a rat model of TKA [28]. In contrast to TXA, which acts downstream of coagulation pathways initiated as a result of surgery-induced inflammation, ALM appears to modulate very early coagulation, inflammatory and central nervous system (CNS) responses, favouring a permissive tissue healing environment [5, 21–23, 28–30]. We hypothesised that compared to TXA, topical administration of ALM during TKA would reduce postoperative coagulopathy and systemic inflammation, and that this would correspond to improved tissue healing in the operated knee. Therefore, using a rat model of knee implant surgery we compared haematology, coagulopathy and systemic inflammation following IA administration of TXA or ALM after joint closure, and before skin closure. In addition, we compared joint capsular healing in the implanted knees, 28 days after surgery.

## Materials and methods

### Animals

Conventional, 20-week-old male Sprague–Dawley rats (350–450 g) were used. Animals were individually housed in ventilated cages (Tecniplast® Australia, NSW, Australia) in a 14–10 h light–dark cycle under controlled temperature (21–22 °C) and humidity (65–75%) conditions, with access to standard rodent pellets (Specialty Feeds, WA, Australia) and water ad libitum.

### Study protocol

**Fig. 1:**
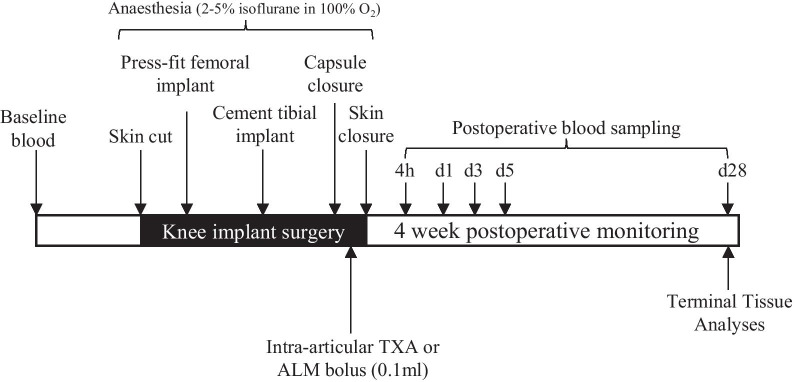
Summary of study design and blood sampling times. Blood was collected from the lateral tail vein of anaesthetised animals for ROTEM, haematology and quantitation of plasma inflammatory cytokines at 4 h, day 1, 3, 5 and 28 postoperative. Animals were euthanised 28 days following surgery for assessment of joint tissue metrics

A total of 24 rats were randomly allocated to two treatment groups, with 12 rats per group. After a 7-day acclimation period, knee implant surgery was performed on animals under isoflurane anaesthesia using surgical techniques and materials described previously [31], and according to the study protocol (Fig. 1). IA injections were administered to the implanted knee immediately following capsule closure, and prior to skin closure as follows: Animals in Group 1 received an IA bolus of TXA (0.1 ml; 25 mg/kg; Juno Pharmaceuticals, South Yarra, VIC), while animals in Group 2 received an IA bolus of ALM (0.1 ml; 1 mM adenosine, 3 mM lidocaine and 2.5 mM MgSO_4_ in 0.9% NaCl). Animals within each treatment group were also randomised to whether end-point tissue samples were to be fixed (histology, *n* = 6 per group) or snap-frozen in liquid nitrogen (gene and protein expression, *n* = 6 per group). Surgeries were performed on 8 animals per surgery day, with equal numbers of animals per treatment group and randomisation of the order of animals undergoing surgery. Intraoperative blood loss was measured as previously described [28]. Clinical signs including body weight, temperature and weight-bearing activity were monitored daily throughout the experimental period. Animals were sacrificed 28 days post-surgery with an overdose of pentobarbital (100 mg/kg) for gross pathology, molecular and histological evaluation.

### Haematology, ROTEM and inflammatory assessments

Blood was collected under anaesthesia via the lateral tail vein during the experimental period or via terminal cardiac puncture at day 28 post-surgery (Fig. 1). Complete blood cell examination (CBE) was carried out using a VetScan HM5 haematology analyser (Abaxis, CA, USA). Rotational thromboelastometry (ROTEM; Tem International, Munich, Germany) was conducted on whole blood according to manufacturer’s instructions. Three 60-min assays were performed simultaneously for each sample: EXTEM (extrinsically activated test using tissue factor), INTEM (intrinsically activated test using ellagic acid) and FIBTEM (EXTEM-activated test with cytochalasin D to inhibit platelet contribution to clot formation). Parameters include clotting time (CT; seconds); alpha angle (*α*°); clot amplitude (mm); maximum clot firmness (MCF, mm); and maximum lysis (%).

Blood samples were centrifuged and plasma collected and stored at − 80 °C until further analysis. Vascular injury markers (adiponectin, sE-selectin, sICAM-1, von Willebrand Factor) were measured in plasma using Milliplex® Rat Vascular Injury Magnetic Bead Panel 2 (Abacus ALS, Meadowbrook, Queensland), in combination with the Magpix® analyser (Luminex Corporation, Austin, Texas, USA). Inflammatory chemokines and cytokines (MIP-1α, IL-1α, IL-1β, MCP-1, TNF-α, IL-6, IL-12p70, IFN-γ, IL-2, IL-4, IL-10, IL-13, RANTES) were measured in plasma and joint tissue homogenates using Milliplex® Rat Cytokine/Chemokine Magnetic Bead Panel (Abacus ALS), as described previously [28].

### Joint angle measurement

The extension range of motion (ROM), or the angle between the longitudinal axis of the femur and the tibia, was measured in triplicate using an angulometer with 20 g force for both the right (implanted) and left (native, control) knee [32]. Data are expressed as the difference in extension ROM between the native and implanted knee from TXA- and ALM-treated animals.

### Tissue collection

Following blood collection at day 28 post-surgery, animals within each treatment arm were sacrificed to assess joint tissue metrics. Macroscopic morphological analysis of the intact joint, the articular surfaces, and capsular and synovial tissue was performed using methods described previously [28]. Briefly, anteromedial capsular tissue samples from the implanted (right) and native (left) knee were dissected, snap-frozen and stored at − 80 °C for subsequent RNA/protein extraction and analysis. Remaining intact knees were fixed in 4% paraformaldehyde (PFA) for routine histology.

### RNA isolation and quantitative RT-PCR of capsular tissue

Total RNA was isolated from anteromedial capsular tissue samples using RNeasy Mini kit (Qiagen) following manufacturer’s protocol. cDNA was prepared by reverse transcription. Real-time PCR with custom-designed primers [28] was used to assess gene expression of nuclear factor kappa B (Nfkb), a key driver of inflammation; and transforming growth factor beta 1 (Tgfb1) and xylosyltransferase (Xylt1), two markers shown to be upregulated in arthrofibrotic tissue [33, 34]. The relative expression of each gene was calculated using the concentration-Ct-standard curve method and normalised using the average expression of the housekeeping ribosomal protein S13 (*Rps13*) gene for each sample [26].

### Histology

For routine histology, fixed intact knees were decalcified (14% EDTA), processed and paraffin-embedded. Sections (4 µm) were cut in the frontal plane, spaced at 50 μm intervals and spanning the entire knee joint [35]. Stained sections were visualised with light microscopy (Nikon Eclipse i50; Japan) and digitised for analysis using ImageJ® software (v1.52p; National Institutes of Health, EUA). Semi-quantitative assessments were carried out by a blinded investigator on five randomly selected fields (400 × magnification) per sections and two sections per joint [28].

Semi-quantitative evaluation of synovitis and fibrotic changes in the joint capsule and infrapatellar fat pad (IFP) was performed on haematoxylin and eosin (H&E) stained sections using methods described previously [28]. Masson–Goldner trichrome staining was used to assess collagen density in the IFP [28]. Using methods described previously [28], mouse anti-rat monoclonal antibodies (Abcam, Cambridge, UK) were used for immunohistochemical localisation of CD68 (ED1, 1:125 dilution) and α-SMA (1A4, 1:1000 dilution). Cells positive for CD68 staining were enumerated in the synovial lining and subsynovial tissue within the medial and lateral capsule of two sections per joint. The percentage of α-SMA-positive staining was assessed in the medial and lateral capsule, and the IFP [28].

### Statistics

Sample sizes (*n*) were determined from a priori power analysis using G-power3 program (Heinrich-Heine-Universität Düsseldorf) using Acta2 gene expression as the outcome measure with effect size Cohen’s *d* = 1.65 (Critical *t* = 1.96; *df* = 11; sample size = 6; *α* = 0.05; calculated power = 0.84). Statistical analyses were performed using GraphPad Prism for Mac software (version 7). Data normality was assessed using Shapiro–Wilks test, with Levene’s test used to determine equality of variances. Independent samples t tests were used for between groups comparison for normally distributed data (haematology parameters, inflammatory marker concentrations). Non-normally distributed data (ROM, macroscopic and histological scores, immunohistochemical staining) were compared using a Mann–Whitney U test. MILLIPLEX Analyst 5.1 software (Luminex Corporation, Austin, Texas, USA) was used to determine cytokine and chemokine concentrations with a 5-parametric logistic weighted curve fit. Results are expressed as mean ± standard error (SEM) unless otherwise stated, with significance set at *p* < 0.05.

## Results

### Effects on surgical metrics and postoperative clinical parameters

There was no significant difference in surgery time between groups (TXA, 17.3 ± 5.9 min vs. ALM, 14.9 ± 3.0 min; *p* = 0.237). Similarly, blood loss during knee implant surgery was comparable for the two treatment groups (TXA, 63.4 ± 26.3 mg vs. ALM, 48.8 ± 35.0 mg; *p* = 0.258). There was also no difference between ALM- and TXA-treated groups for the mean total time spent under isoflurane anaesthesia in the first week following surgery (TXA, 124 ± 19 min vs. ALM, 120 ± 13 min; *p* = 0.576). Following surgery, the time taken to regain preoperative body weight (TXA, 19 ± 4 d vs. ALM, 19 ± 5 d; *p* = 0.935) and full weight-bearing in the operated limb (TXA, 7 ± 5 d vs. ALM, 7 ± 3 d; *p* = 0.748) was similar for TXA- and ALM-treated animals.

### Effect on coagulopathy

Surgery-induced coagulopathy was evident in both treatment groups indicated by significantly reduced clot amplitudes (A10, MCF) and clot kinetics (α angle) on EXTEM and INTEM tests at 4 h and day 1, with return to baseline levels by day 3 post-surgery (Additional file 1: Table S1). Animals in the TXA group had increased clot times and reduced clot stability compared to ALM-treated animals in the early postoperative period, though differences were not statistically significant. There was no evidence of hyperfibrinolysis (ML > 15%) in either group (Additional file 1: Table S1).

### Effect on haematology

**Fig. 2:**
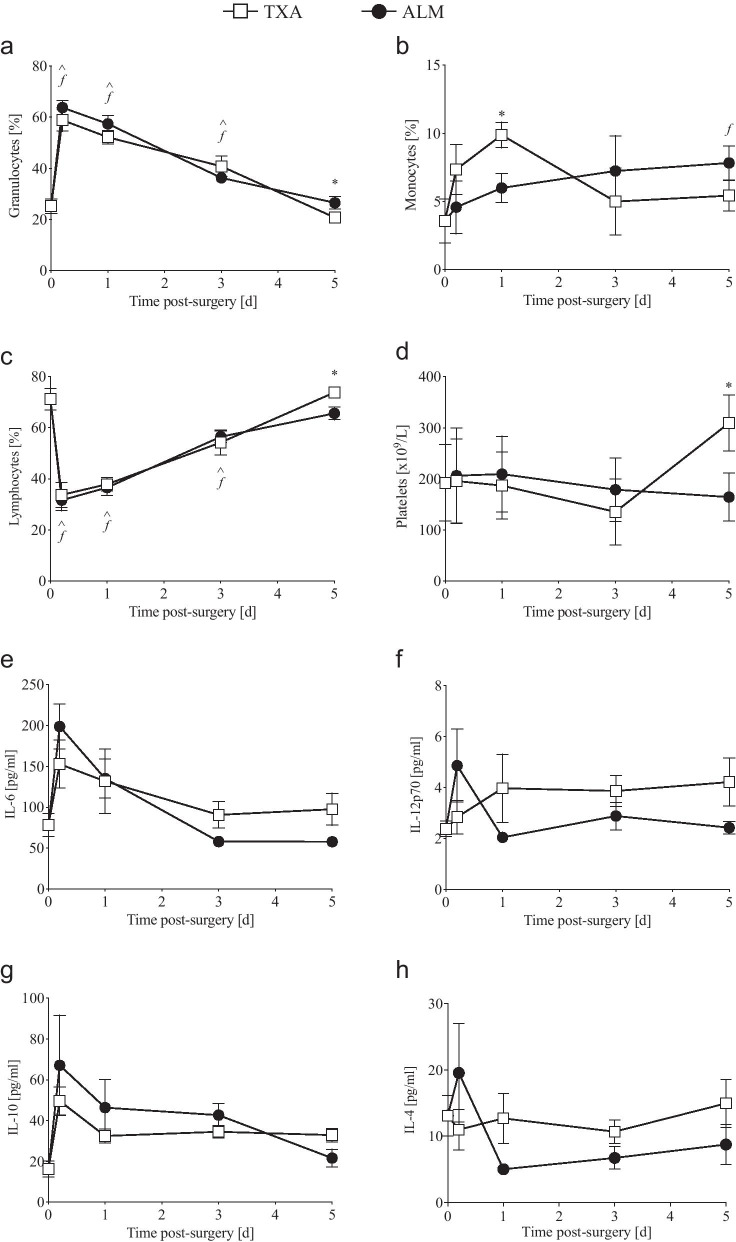
Changes in early postoperative haematology and systemic inflammatory cytokines. **a–c** Percentage (%) of circulating granulocytes, monocytes and lymphocytes, **d** platelet numbers and **e–h** plasma concentrations of IL-6, IL-12p70, IL-10 and IL-4 for TXA- and ALM-treated animals at 4 h, day 1, 3 and 5 postoperative. Data represent mean ± SEM. ^* p* < 0.05 TXA compared to Baseline. ^ƒ^*p* < 0.05 ALM compared to Baseline. *p < 0.05 TXA compared to ALM

Total leucocyte numbers remained comparable to baseline levels following surgery, with no significant difference observed between treatment groups to day 5 (Additional file 2: Table S2). Compared to baseline levels, the number and percentage of circulating granulocytes increased significantly 4 h after surgery, before gradually returning to preoperative levels by day 5 post-surgery (Fig. 2a; Additional file 2: Table S2). However, the percentage of granulocytes was higher in ALM-treated animals at day 5 post-surgery, compared to TXA-treated animals (*p* = 0.04; Fig. 2a). Compared to baseline and ALM-treated animals, circulating monocyte numbers (*p* = 0.04 and ns, *p* = 0.09, respectively) and percentages of monocytes (*p* = 0.009 and *p* = 0.022, respectively) were increased in TXA-treated animals at day 1 postoperative (Fig. 2b; Additional file 2: Table S2). However, while the monocyte percentage returned to baseline levels by day 3 in animals that received TXA, a steady rise in monocytes was observed in ALM-treated animals, with levels significantly higher than baseline at day 5 (*p* = 0.049; Fig. 2b). Following surgery, the percentage of circulating lymphocytes remained significantly lower than baseline levels to day 3 for animals in both treatment groups (Fig. 2c; Additional file 2: Table S2). While the percentage of circulating lymphocytes returned to baseline in TXA-treated animals by day 5 post-surgery, levels remained significantly lower in ALM-treated animals (*p* = 0.019; Fig. 2c).

Following surgery, minor decreases were observed in haematocrit and haemoglobin levels from 4 h to day 5 postoperative, however changes were comparable between treatment groups (Additional file 2: Table S2). Platelet numbers remained comparable to preoperative levels to day 3 post-surgery for both TXA- and ALM-treated animals (Fig. 2d). At day 5 following surgery however, platelet numbers were 1.6-fold higher than baseline in the TXA group, and significantly higher than ALM-treated animals (*p* = 0.024; Fig. 2d).

### Effect on systemic inflammation

#### Vascular injury markers

Compared to preoperative levels (8.1 ± 1.5 ng/ml), the plasma concentration of sICAM, a marker of vascular inflammation, was significantly increased in TXA-treated animals at 4 h (12.4 ± 2.7 ng/ml; *p* = 0.009) and day 1 (11.0 ± 1.2 ng/ml; *p* = 0.009) postoperative. In contrast, sICAM concentrations in ALM-treated animals at 4 h (10.5 ± 6.1 ng/ml; *p* = 0.662) and day 1 (9.6 ± 4.9 ng/ml; *p* = 0.93) postoperative remained comparable to baseline levels. Other vascular injury markers (adiponectin, sE-selectin, vWF) were significantly elevated at 4 h and day 1, however no differences were observed between TXA- and ALM-treated animals (Additional file 2: Table S2).

#### Inflammatory cytokines and chemokines

At 4 h post-surgery, plasma levels of IL-6 increased significantly from baseline in ALM-treated animals, returning to pre-surgical levels by day 3 (Fig. 2e). Systemic IL-6 did not differ between treatment groups up to 3 days postoperative. However, IL-6 levels were 1.7-fold lower in ALM-treated animals at day 5 following surgery (*p* = 0.054; Fig. 2e). Similar trends were observed for IL-12p70 (*p* = 0.08), IL-10 (*p* = 0.051) and IL-4 (*p* = 0.203), with higher concentrations in the TXA group at day 5 postoperative, compared to ALM-treated animals (Fig. 2f–h). No significant differences were observed in systemic MCP-1, IL-1β or IFN-γ following surgery, with levels comparable for TXA- and ALM-treated animals at all timepoints investigated (Additional file 2: Table S2). TNF-α was not detected at any time postoperative (Additional file 2: Table S2).

Compared to preoperative baseline concentrations, plasma levels of IL-1β and IL-10 remained significantly elevated in TXA-treated animals, 4 weeks after knee implant surgery (Fig. 4a, b). In contrast, preoperative and 28-day postoperative systemic inflammatory cytokine levels in ALM-treated animals were comparable for all inflammatory markers assessed (Additional file 2: Table S2).

### Effects on implanted joint

#### Macroscopic changes

**Fig. 3:**
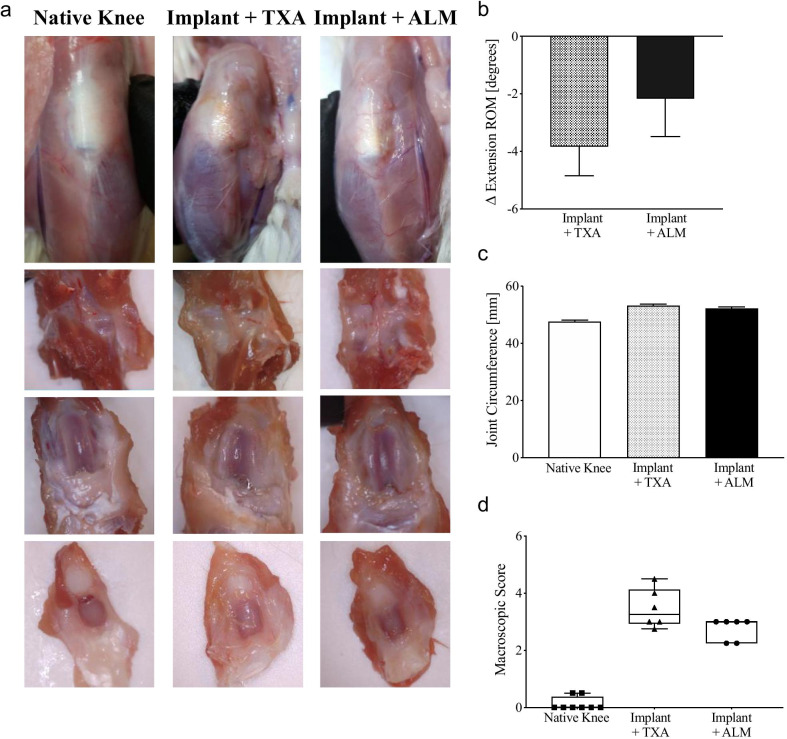
Macroscopic pathology of implanted knees. Macroscopic changes in implanted knees at day 28 following surgery in animals that received an IA bolus of TXA or ALM. **a** Representative images of a native knee and implanted knees from TXA- and ALM-treated animals. External views of the knee joints after removal of the skin (upper panels). Posterior (second row) and internal (third row) view of the joints following dissection of the patella (lower panels). **b** Differences in the extension range of motion (ROM) for native and implanted knees tended to be greater for TXA- compared to ALM-treated animals. **c** Compared to native knees, the circumference of implanted knees was increased for TXA- and ALM-treated animals. **d** Macroscopic pathology scores tended to be lower for ALM- than TXA-treated knees. Data show mean ± SEM

Macroscopically, mild-to-moderate synovial and capsular tissue hyperplasia and cartilage defects were evident in implanted knees (Fig. 3a). While gross pathology scores tended to be lower for ALM- than TXA-treated knees, these differences were not statistically significant (*p* = 0.108; Fig. 3a, d). Similarly, the loss of extension ROM at 4 weeks postoperative tended to be greater for TXA-treated knees (-3.8 ± 3.5 degrees), than those administered ALM (-2.2 ± 4.6 degrees) (*p* = 0.243; Fig. 3b). While some joint swelling was evident in implanted knees after 4 weeks compared to native knees, there was no significant difference between treatment groups (*p* = 0.273; Fig. 3c).

#### Inflammation

**Fig. 4:**
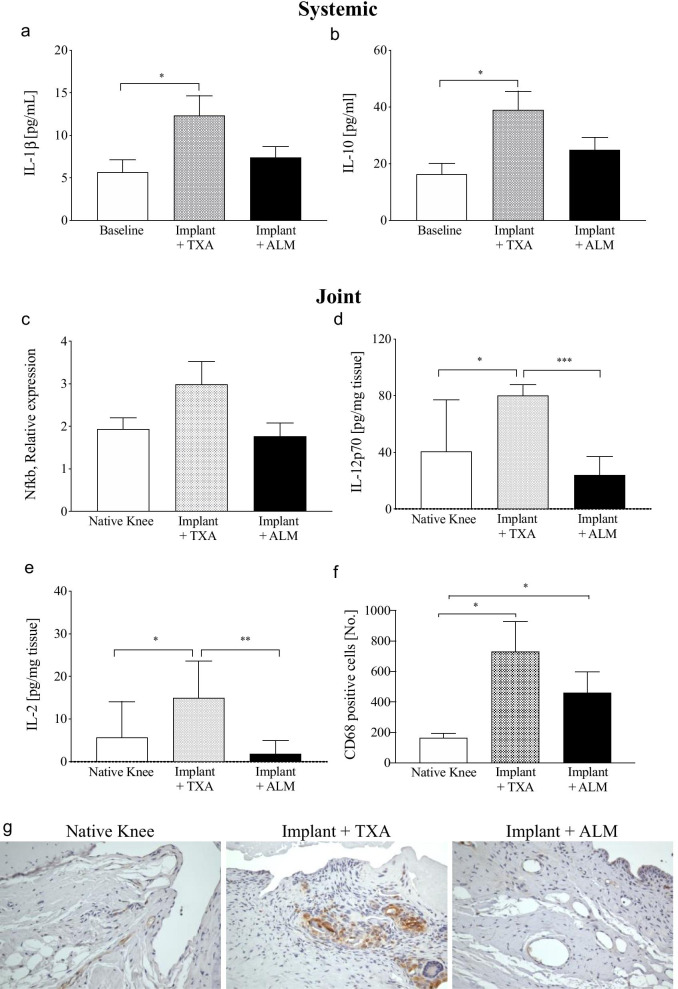
Persistent systemic and local joint inflammation. Levels of **a** IL-1β and **b** IL-10 in plasma and **c** NF-κB, **d** IL-12p70 and **e** IL-2 in joint capsular tissue were higher for TXA- than ALM-treated animals at day 28 following surgery. **f** Compared to TXA-treated knees, numbers of CD68-positive macrophages were lower in capsular tissue from knees administered an IA bolus of ALM at day 28 following surgery. **g** Representative images of CD68 staining in medial capsular tissue of native and implanted knees at day 28 following surgery (magnification, × 100). Data show mean ± SEM. *p < 0.05, **p < 0.01, ***p < 0.001, compared to TXA

Expression of NF-κB, a key modulator of inflammation, tended to be higher in joint capsular tissue of TXA-treated knees compared to native (*p* = 0.064) and ALM-treated knees (*p* = 0.052; Fig. 4c). Levels of IL-12p70 and IL-2 were also significantly higher in TXA-treated knees compared to native (*p* = 0.027 and *p* = 0.043, respectively) and ALM-treated knees (*p* < 0.001 and *p* = 0.006, respectively; Fig. 4d, e). Capsular tissue concentrations of the other inflammatory cytokines and chemokines assessed were comparable between treatment groups (Additional file 3: Table S3). Compared to native knees, the mean number of CD68-positive cells was increased in the capsular tissue from implanted knees (TXA, 4.5-fold; ALM, 2.8-fold; Fig. 4f, g). Consistent with the disparate pro-inflammatory cytokines levels, a 1.6-fold decrease was observed in the number of CD68-positive cells within the synovium and subsynovial capsular tissue of ALM-treated knees, compared to TXA-treated knees (*p* = 0.31; Fig. 4f, g).

#### Fibrotic changes and ECM remodelling

**Fig. 5:**
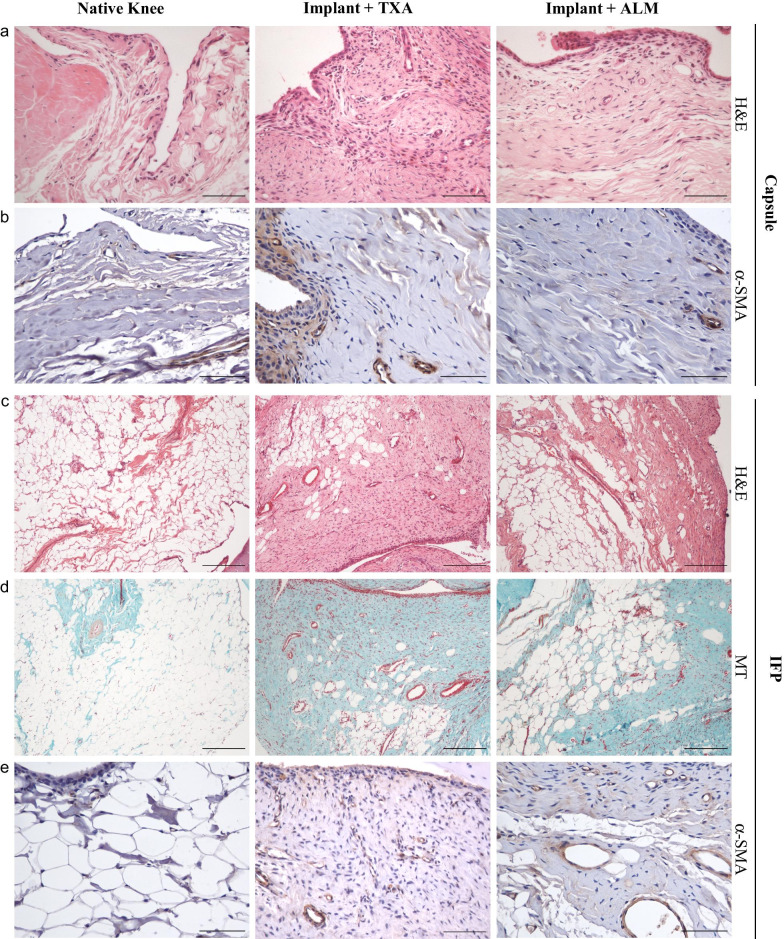
Histopathology of joint capsular tissue and infrapatellar fat pad (IFP). Representative stained sections of anteromedial joint capsular tissue and infrapatellar fat pad (IFP) of native, TXA- and ALM-treated knees at day 28 following surgery. **a, c** Haematoxylin and eosin (H&E), **b, e** α-SMA and **d** Masson–Goldner trichrome staining (MT) (magnification, × 100 and × 400; scale bar, 0.5 mm)

**Fig. 6:**
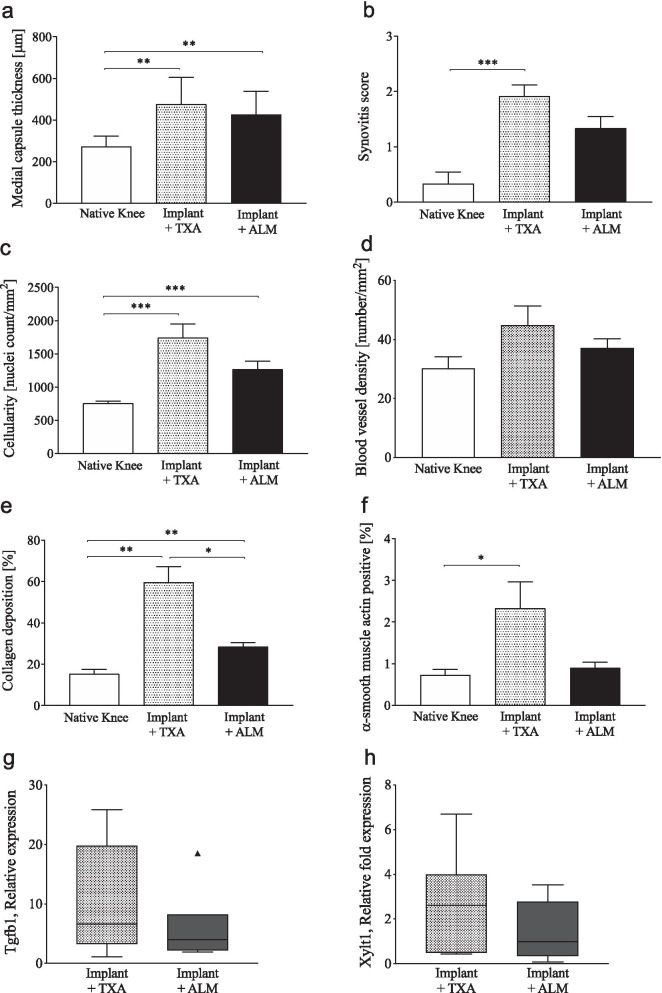
Joint tissue remodelling and fibrosis. Despite similarities in **a** medial capsule thickness, **b** synovitis, **c** cellularity, **d** blood vessel density, **e** collagen deposition and **f** α-SMA-positive staining tended to be lower within joint capsule tissue from ALM-treated knees, compared to TXA-treated knees at day 28 following surgery. mRNA expression of the fibrotic markers, Tgfb1 and Xylt1, also tended to be decreased in joint capsular tissue of ALM-treated knees. Data show mean ± SEM, *p < 0.05, **p < 0.01, ***p < 0.001

Histological comparison of capsular tissue and IFP of implanted knees in the TXA- and ALM-treatment groups, as well as native knees was performed to examine pathological changes in joint tissue architecture 4 weeks following implant surgery (Figs. 5, 6). Thickness of the medial joint capsule was increased in implanted knees (TXA, 1.7-fold, *p* = 0.001; ALM, 1.6-fold, *p* = 0.004), compared to native knees (Fig. 6a). Synovitis was increased in TXA-treated, implanted knees compared to native knees (*p* = 0.003; Figs. 5, 6b). Implanted knees of ALM-treated animals exhibited less synovitis than TXA-treated animals, though these differences were not statistically significant (*p* = 0.178; Figs. 5, 6b). Compared to native knees, cellularity was increased in the capsular tissue of implanted knees (TXA, *p* < 0.001; ALM, *p* < 0.001; Fig. 6c). At 4 weeks following surgery, cellularity was 1.4-fold lower in joint capsular tissue from ALM- compared to TXA-treated knees (*p* = 0.076; Fig. 6c). No significant differences were observed in blood vessel density within joint capsule tissue between treatment groups (Fig. 6d), however subsynovial collagen deposition was greater in joint capsule tissue and IFP of TXA-treated animals, compared to those treated with ALM (*p* = 0.041; Figs. 5, 6e). Similarly, the percentage of the myofibroblast marker α-SMA-positive staining was increased in capsular tissue and IFP from TXA-treated animals, compared to native (*p* = 0.035) and ALM-treated, implanted knees (*p* = 0.055; Figs. 5, 6f). mRNA expression of fibrosis markers, Tgfb1 and Xylt1, also tended to be lower in joint capsule tissue of implanted knees treated with ALM, than those administered TXA, however differences were not statistically significant (*p* = 0.39 and *p* = 0.31, respectively; Fig. 6g, h).

## Discussion

TXA use has been increasingly adopted as a blood management and possible immunomodulatory strategy in TKA, despite ongoing controversies regarding its safety and long-term effects on joint tissues [6, 17, 19, 36]. A new ALM drug therapy is being developed to treat traumatic injury, and topical administration of ALM was recently shown to reduce arthrofibrosis following experimental TKA [28]. Here, we report that a single IA bolus of ALM delivered after experimental knee implant surgery, and prior to skin closure, reduced systemic inflammation in the early postoperative period compared to TXA. ALM was also associated with improved restoration of healthy joint tissue architecture and increased ROM in implanted knees, 4 weeks after surgery. There was a more pronounced post-surgical hypocoagulopathy in the TXA group, with no hyperfibrinolysis. These results will now be discussed.

### Systemic effects of TXA and ALM on postoperative bleeding, coagulopathy and hyperfibrinolysis

Mild acute blood loss anaemia occurred in both groups in the first 5 days following surgery, indicated by a temporary decrease in haematocrit (~ 9%) and haemoglobin (~ 15%), and consistent with the comparable postoperative blood loss measured in both groups (Additional file 2: Table S2). Despite similar blood loss, ROTEM analysis indicated differences in coagulopathy. Both groups had increased CT, decreased clot amplitude and firmness after 4 h and day 1. However, EXTEM clot time in the TXA group was higher than baseline at 4 h (twofold increase) and day 1 (1.7-fold increase) indicating a more pronounced hypocoagulopathy early after surgery. This TXA ‘slowing effect’ on coagulation time is consistent with our previous findings for TKA patients [15]. The absence of hyperfibrinolysis after TKA also supports our previous human study [15]. Both studies highlight the need for clinical justification using a drug like TXA without first establishing a need. For example, while numerous studies have demonstrated the effectiveness of IV and IA TXA administration to reduce perioperative blood loss and haemarthrosis following orthopaedic procedures [8–10, 12], others have reported an increased risk of venous thromboembolism, particularly for patients with contraindications such as cardiovascular disease or previous myocardial infarction [12, 36]. IA administration of TXA is increasingly cited as a preferred route, producing comparable postoperative systemic coagulation and fibrinolytic profiles to systemic administration [37]. However, there is controversial evidence of TXA chondrotoxicity following topical administration [17, 18, 36, 38]. Given the ongoing controversies, we believe current guidelines should address the indiscriminate use of TXA and recommend against its widespread use in major surgery, like TKA, without first demonstrating the presence of hyperfibrinolysis.

### Differential effects of TXA and ALM on systemic inflammation

Our study also showed that plasma inflammatory cytokines remained elevated in TXA-treated animals at day 5 (IL-6, IL-12, IL-10) and day 28 (IL-1β, IL-10) following surgery. In contrast, following a rapid, early rise in plasma inflammatory cytokines in the ALM group, concentrations of these mediators returned to preoperative levels by day 3. Systemic inflammation is important for optimal healing and functional recovery after trauma, however when delayed or overexpressed inflammation can lead to slow healing and organ dysfunction [5]. The rapid return of systemic inflammatory mediators to baseline by day 3 in ALM-treated animals indicates a more optimal healing environment for tissue recovery compared to TXA-treated animals.

The increased systemic inflammatory response in the TXA group was also associated with an early rapid rise in peripheral blood granulocytes and monocytes, with levels returning to baseline by day 5 (Fig. 2, Additional file 2: Table S2). In direct contrast, the percentage of granulocytes in the ALM group remained significantly elevated at day 5, and circulating monocytes rose from 4 h to day 5. The early return of granulocytes and monocytes to baseline in TXA-treated animals is intriguing and may be due to TXA’s effect to inhibit plasmin, which is a potent facilitator of granulocyte and monocyte migration to sites of inflammation [39, 40]. This was not the case in the ALM-treated animals and may reflect differences in the effect of TXA and ALM on the initiation and progression of inflammatory and immune responses within the implanted knee (see below) [5].

Another interesting finding was that platelet numbers were significantly higher in TXA than ALM-treated animals at day 5, which is consistent with persistent systemic inflammation in the former group [41], though reasons for this increase are unclear. The anti-plasmin effect of TXA is known to reduce platelet function, however platelet function was not assessed in the present study. These data, together with the findings of Barret et al. [42] who showed that TXA can have varied effects on inflammation depending on the dose and route of administration (IV or topical), warrant further mechanistic studies of TXA’s dual inflammatory actions in different trauma contexts, and give reason for caution against the universal adoption of TXA in orthopaedic procedures, particularly in the absence of hyperfibrinolysis.

### ALM decreases joint inflammation and promotes tissue healing

Two standout findings in the present study were: 1) the persistence of systemic inflammation in TXA-treated animals appears to be due to factors (e.g. DAMPs) released from the inflamed implanted joint itself and 2) inflammation was reduced in ALM-treated knees, which was supported by decreased expression of NF-κB, IL-12 and IL-2 in capsular tissue, compared to the TXA group (Fig. 4). NF-κB is an inducible transcription factor and its overexpression plays a central role in secondary damage progression by activating inflammatory and immune responses, and abnormal tissue remodelling. In addition, ALM protection is consistent with histological findings of decreased synovitis, reduced mononuclear leucocyte infiltration, and fewer cells expressing CD68, predominantly tissue macrophages, in joint capsular tissue from ALM-treated, implanted knees. While it was beyond the scope of the current study to determine the complete composition of the subsynovial immune cell infiltrate, previous studies [43] show a predominance of T lymphocytes in arthrofibrotic tissue, which is consistent with the elevated IL-2 detected in capsular tissue from TXA-treated knees in the current study.

Sustained local inflammation following TKA drives the accumulation of fibrotic and collagenous tissue within the knee, resulting in restricted joint movement and pain [2, 44]. Our study showed that animals administered an IA bolus of ALM had a 1.7-fold improvement in extension ROM of the operated knee, which at 28 days was associated with a reduction in myofibroblasts, decreased expression of fibrosis-associated markers, TGF-β and xylosyltransferase I, and a reduction in collagenous matrix density in the IFP and joint capsular tissue (Figs. 5, 6). In contrast to TXA, cellular organisation of joint capsular tissue in implanted knees administered ALM more closely resembled healthy tissue, with the anti-inflammatory and anti-fibrotic protection afforded by ALM extending to 4 weeks postoperative [24, 26]. In summary, compared to TXA which appears to impair leucocyte mobilisation and promote persistent local and systemic inflammation, IA administration of ALM appears to facilitate early inflammatory responses with accelerated progression to a healing phenotype within the implanted knee. The mechanisms underlying this ALM-induced healing phenotype are yet to be elucidated.

### Limitations

A potential limitation of experimental models of TKA is that the pre-existing comorbidities in many patients undergoing TKA are not reflected as most use healthy animals. Nevertheless, the rat TKA model exerts systemic and local inflammatory responses that are comparable to those reported in the clinical setting [45, 46] and joint fibrosis after 4 weeks [28], making it a useful preclinical model for drug evaluation. A second potential limitation of the present study is that we did not evaluate different concentrations of TXA. However, the single IA dose of 25 mg/kg administered into the joint capsule, prior to skin closure is within the clinical TXA dose range of 1.5 g to 3 g, equivalent to ~ 12 to 35 mg/kg based on an average male weight of 86 kg [7, 9, 12, 14, 47]. Therefore, we believe the significant elevation in postoperative inflammation and worse pathology after topical TXA use may have clinical relevance.

### Potential clinical relevance

Post-TKA arthrofibrosis is a significant problem [2, 48, 49]. *Current therapies are limited and tend to be single nodal therapies that address symptoms, not the underlying causes of inflammation and fibrosis.* The present study showing that a single IA injection of ALM led to reduced systemic and joint inflammation with improved tissue histology and increased ROM, is encouraging for future translational studies from animals to humans. It is possible that ALM may fill current gaps in treatment during and after TKA surgery to reduce stiffness and arthrofibrosis, and possibly pain [28]. To this end, therapeutic concentrations of ALM have also been shown to be protective to human chondrocytes [27].

## Conclusion

In conclusion, this study demonstrated that compared to TXA, IA administration of ALM after capsule closure, and before skin closure in an experimental model of knee implant surgery, was associated with a significant reduction in early postoperative systemic and joint inflammation, with improved joint tissue healing and increased ROM. Data suggest that local delivery of TXA may alter the temporal progression of local and systemic immune responses and delay tissue repair processes. ALM may be a frontline strategy to promote a permissive healing environment within the implanted knee and improve patient outcomes following TKA.

## Supplementary Information

**Additional file 1: Table S1**. Postoperative ROTEM parameters.

**Additional file 2: Table S2**. Postoperative clinical, haematology and systemic inflammatory parameters.

**Additional file 3: Table S3**. Inflammatory cytokines in joint capsular tissue.

## Data Availability

All data pertaining to the present study have been included in this manuscript. The authors are willing to share the raw data upon reasonable request to the corresponding author.

## Abbreviations

ACL: Anterior cruciate ligament
ALM: Adenosine, lidocaine and Mg^2^^+^
α-SMA: Alpha smooth muscle actin
CCN2: Connective tissue growth factor
ECM: Extracellular matrix
EDTA: Ethylenediaminetetraacetic acid
FGF: Fibroblast growth factor
H&E: Haematoxylin and eosin
IA: Intra-articular
IFP: Infrapatellar fat pad
IL: Interleukin
MT: Masson–Goldner trichrome staining
NFκB: Nuclear factor kappa b
PFA: Paraformaldehyde
RANTES: Regulated upon activation, normal T cell expressed and presumably secreted
RNA: Ribonucleic acid
ROM: Range of motion
ROTEM: Rotational thromboelastometry
SD: Standard deviation
sICAM: Soluble intercellular adhesion molecule
TGFβ: Transforming growth factor beta
TKA: Total knee arthroplasty
UHXLPE: Ultra-highly cross-linked polyethylene
vWF: Von Willebrand factor
XYLT: Xylosyltransferase

## References

[1] Singh JA, Yu S, Chen L, Cleveland JD (2019). Rates of total joint replacement in the United States: future projections to 2020–2040 using the National Inpatient Sample. J Rheumatol.

[2] Thompson R, Novikov D, Cizmic Z, Feng JE, Fideler K, Sayeed Z (2019). Arthrofibrosis after total knee arthroplasty: pathophysiology, diagnosis, and management. Orthop Clin North Am.

[3] Tibbo ME, Limberg AK, Salib CG, Ahmed AT, Wijnen AJ, Berry DJ (2019). Acquired idiopathic stiffness after total knee arthroplasty: a systematic review and meta-analysis. J Bone Jt Surg Am.

[4] Malahias MA, Birch GA, Zhong H, Sideris A, Gonzalez Della Valle A, Sculco PK (2020). Postoperative serum cytokine levels are associated with early stiffness after total knee arthroplasty: a prospective cohort study. J Arthroplasty.

[5] Dobson GP, Biros E, Letson HL, Morris JM (2021). Living in a hostile world: inflammation, new drug development, and coronavirus. Front Immunol.

[6] Danninger T, Memtsoudis SG (2015). Tranexamic acid and orthopedic surgery-the search for the holy grail of blood conservation. Ann Transl Med.

[7] Fillingham YA, Ramkumar DB, Jevsevar DS, Yates AJ, Bini SA, Clarke HD (2018). Tranexamic acid use in total joint arthroplasty: the clinical practice guidelines endorsed by the American Association of Hip and Knee Surgeons, American Society of Regional Anesthesia and Pain Medicine, American Academy of Orthopaedic Surgeons, Hip Society, and Knee Society. J Arthroplasty.

[8] La Banca V, Suzuki Leal Roque JG, Protta T, Schmidt Navarro M (2021). Evaluation of antifibrinolytic use in anterior cruciate ligament arthroscopic reconstruction: a prospective clinical trial. Muscles Ligaments Tendons J.

[9] Abdel MP, Chalmers BP, Taunton MJ, Pagnano MW, Trousdale RT, Sierra RJ (2018). Intravenous versus topical tranexamic acid in total knee arthroplasty: both effective in a randomized clinical trial of 640 patients. J Bone Jt Surg Am.

[10] Demos HA, Lin ZX, Barfield WR, Wilson SH, Robertson DC, Pellegrini VD (2017). Process improvement project using tranexamic acid is cost-effective in reducing blood loss and transfusions after total hip and total knee arthroplasty. J Arthroplasty.

[11] Karaaslan K, Karaoğlu S, Yurdakul E (2015). Reducing intra-articular hemarthrosis after arthroscopic anterior cruciate ligament reconstruction by the administration of intravenous tranexamic acid: a prospective, randomized controlled trial. Am J Sports Med.

[12] Moskal JT, Capps SG (2018). Intra-articular tranexamic acid in primary total knee arthroplasty: meta-analysis. J Knee Surg.

[13] Tille E, Mysliwietz J, Beyer F, Postler A, Lützner J (2019). Intraarticular use of tranexamic acid reduces blood loss and transfusion rate after primary total knee arthroplasty. BMC Musculosk Disord.

[14] Goyal N, Chen DB, Harris IA, Rowden NJ, Kirsh G, MacDessi SJ (2017). Intravenous vs intra-articular tranexamic acid in total knee arthroplasty: a randomized, double-blind trial. J Arthroplasty.

[15] Grant AL, Letson HL, Morris JL, McEwen P, Hazratwala K, Wilkinson M (2018). Tranexamic acid is associated with selective increase in inflammatory markers following total knee arthroplasty (TKA): a pilot study. J Orthop Surg Res.

[16] Jang B, Kao M, Bohm MT, Harris IA, Chen DB, MacDessi SJ (2014). Intra-articular injection of tranexamic acid to reduce blood loss after total knee arthroplasty. J Orthop Surg (Hong Kong).

[17] Parker JD, Lim KS, Kieser DC, Woodfield TBF, Hooper GJ (2018). Is tranexamic acid toxic to articular cartilage when administered topically? What is the safe dose?. Bone Joint J.

[18] Bolam SM, O’Regan-Brown A, Monk AP, Musson DS, Cornish J, Munro JT (2020). Toxicity of tranexamic acid (TXA) to intra-articular tissue in orthopaedic surgery: a scoping review. Knee Surg Sports Traumatol Arthrosc.

[19] McLean M, McCall K, Smith IDM, Blyth M, Kitson SM, Crowe LAN (2019). Tranexamic acid toxicity in human periarticular tissues. Bone Joint Res.

[20] Davenport L, Letson HL, Dobson GP (2017). Immune-inflammatory activation after a single laparotomy in a rat model: effect of adenosine, lidocaine and Mg2+ infusion to dampen the stress response. Innate Immun.

[21] Dobson GP, Letson HL. Technical report: a new ultra-small volume fluid for far-forward, non-compressible hemorrhage and traumatic brain injury. Fort Detrick, Maryland; 2016. https://apps.dtic.mil/sti/citations/AD1007400.

[22] Dobson GP (2020). Trauma of major surgery: a global problem that is not going away. Int J Surg.

[23] Dobson GP, Letson HL (2020). Far forward gaps in hemorrhagic shock and prolonged field care: an update of ALM fluid therapy for field use. J Spec Oper Med.

[24] Letson HL, Dobson GP (2017). Adenosine, lidocaine and Mg2+ (ALM) fluid therapy attenuates systemic inflammation, platelet dysfunction and coagulopathy after non-compressible truncal hemorrhage. PLoS ONE.

[25] Letson HL, Dobson GP (2017). 3% NaCl adenosine, lidocaine, Mg2+ (ALM) bolus and 4 hours “drip” infusion reduces noncompressible hemorrhage by 60% in a rat model. J Trauma Acute Care Surg.

[26] Letson HL, Morris JL, Biros E, Dobson GP (2019). Adenosine, lidocaine, and Mg2+ fluid therapy leads to 72-hour survival after hemorrhagic shock: A model for studying differential gene expression and extending biological time. J Trauma Acute Care Surg.

[27] McCutchan A, Dobson GP, Stewart N, Letson HL, Grant AL, Jovanovic IA (2019). Absence of cytotoxic and inflammatory effects following in vitro exposure of chondrogenically-differentiated human mesenchymal stem cells to adenosine, lidocaine and Mg2+ solution. J Exper Orthop.

[28] Morris JL, Letson HL, McEwen P, Biros E, Dlaska C, Hazratwala K (2021). Intra-articular adenosine, lidocaine and magnesium (ALM) solution decreases postoperative joint fibrosis in an experimental knee implant model. Transl Med Commun.

[29] Dobson GP, Letson HL (2016). Adenosine, lidocaine, and Mg2+ (ALM): From cardiac surgery to combat casualty care—teaching old drugs new tricks. J Trauma Acute Care Surg.

[30] Dobson GP, Morris JL, Davenport L, Letson HL (2020). Traumatic-induced coagulopathy as a systems failure: a new window into hemostasis. Semin Thromb Hemost.

[31] Morris JL, Letson HL, Grant A, Wilkinson M, Hazratwala K, McEwen P (2019). Experimental model of peri-prosthetic infection of the knee caused by *Staphylococcus aureus* using biomaterials representative of modern TKA. Biol Open.

[32] Gao ZY, Wu JX, Liu WB, Sun JK (2017). Reduction of adhesion formation after knee surgery in a rat model by botulinum toxin A. Biosci Rep.

[33] Bernstein A, Reichert SNA, Südkamp NP, Hernandez SL, Nerlich AG, Kühle J (2020). Expression of xylosyltransferases I and II and their role in the pathogenesis of arthrofibrosis. J Orthop Surg Res.

[34] Faust I, Traut P, Nolting F, Petschallies J, Neumann E, Kunisch E (2015). Human xylosyltransferases—mediators of arthrofibrosis? New pathomechanistic insights into arthrofibrotic remodeling after knee replacement therapy. Sci Rep.

[35] Gerwin N, Bendele AM, Glasson S, Carlson CS (2010). The OARSI histopathology initiative—recommendations for histological assessments of osteoarthritis in the rat. Osteoarthr Cartil.

[36] Siegel MG (2019). The dangers and concerns of intra-articular tranexamic acid. Arthrosc.

[37] Jules-Elysee KM, Tseng A, Sculco TP, Baaklini LR, McLawhorn AS, Pickard AJ (2019). Comparison of topical and intravenous tranexamic acid for total knee replacement: a randomized double-blinded controlled study of effects on tranexamic acid levels and thrombogenic and inflammatory marker levels. J Bone Joint Surg Am.

[38] Tuttle JR, Feltman PR, Ritterman SA, Ehrlich MG (2015). Effects of tranexamic acid cytotoxicity on in vitro chondrocytes. Am J Orthop (Belle Mead NJ).

[39] Reichel CA, Lerchenberger M, Uhl B, Rehberg M, Berberich N, Zahler S (2011). Plasmin inhibitors prevent leukocyte accumulation and remodeling events in the postischemic microvasculature. PLoS ONE.

[40] Silva LM, Lum AG, Tran C, Shaw MW, Gao Z, Flick MJ (2019). Plasmin-mediated fibrinolysis enables macrophage migration in a murine model of inflammation. Blood.

[41] Stokes KY, Granger DN (2012). Platelets: a critical link between inflammation and microvascular dysfunction. J Physiol.

[42] Barrett CD, Moore HB, Kong YW, Chapman MP, Sriram G, Lim D (2019). Tranexamic acid mediates proinflammatory and anti-inflammatory signaling via complement C5a regulation in a plasminogen activator–dependent manner. J Trauma Acute Care Surg.

[43] Bosch U, Zeichen J, Skutek M, Haeder L, van Griensven M (2001). Arthrofibrosis is the result of a T cell mediated immune response. Knee Surg Sports Traumatol Arthrosc.

[44] Usher KM, Zhu S, Mavropalias G, Carrino JA, Zhao J, Xu J (2019). Pathological mechanisms and therapeutic outlooks for arthrofibrosis. Bone Res.

[45] Langkilde A, Jakobsen TL, Bandholm TQ, Eugen-Olsen J, Blauenfeldt T, Petersen J (2017). Inflammation and post-operative recovery in patients undergoing total knee arthroplasty-secondary analysis of a randomized controlled trial. Osteoarthr Cartil.

[46] Si HB, Yang TM, Zeng Y, Zhou ZK, Pei FX, Lu YR (2017). Correlations between inflammatory cytokines, muscle damage markers and acute postoperative pain following primary total knee arthroplasty. BMC Musculoskelet Disord.

[47] Fryar CD, Kruszon-Moran D, Gu Q, Ogden CL (2018). Mean body weight, height, waist circumference, and body mass index among adults: United States, 1999–2000 through 2015–2016. Natl Health Stat Rep.

[48] Abdul N, Dixon D, Walker A, Horabin J, Smith N, Weir DJ (2015). Fibrosis is a common outcome following total knee arthroplasty. Sci Rep.

[49] Chen AF, Lee YS, Seidl AJ, Abboud JA (2019). Arthrofibrosis and large joint scarring. Connect Tissue Res.

