# Supplementary material for: Comparison of intra-articular administration of adenosine, lidocaine and magnesium solution and tranexamic acid for alleviating postoperative inflammation and joint fibrosis in an experimental model of knee arthroplasty
Source: J Orthop Surg Res. 2021 Dec 20;16:726. doi: 10.1186/s13018-021-02871-y (PMC8686251; doi:10.1186/s13018-021-02871-y)
Supplement: Supplementary file 1 — Additional file 1: Table S1. Postoperative ROTEM parameters. [file 13018_2021_2871_MOESM1_ESM.docx]

**Table S1.** ROTEM parameters at 4, 1 and 3 days following surgery.

| **Parameter** | | **Baseline** | **Time** | **TXA** | **ALM** |
| --- | --- | --- | --- | --- | --- |
| CT, sec | EXTEM | 41 ± 2 | 4h | 82 ± 30* | 61 ± 9 |
|  |  |  | day 1 | 70 ±10* | 62 ± 8* |
|  |  |  | day 3 | 62 ± 14* | 57 ± 15 |
|  | INTEM | 82 ± 9 | 4h | 131 ± 42* | 116 ± 19 |
|  |  |  | day 1 | 148 ± 37* | 176 ± 94 |
|  |  |  | day 3 | 122 ± 51 | 122 ± 34 |
|  | FIBTEM | 39 ± 3 | 4h | 76 ± 37 | 56 ± 11 |
|  |  |  | day 1 | 98 ± 75 | 62 ± 13* |
|  |  |  | day 3 | 67 ± 50 | 55 ± 11* |
| α, ^o^ | EXTEM | 83 ± 1 | 4h | 66 ± 8* | 66 ± 14* |
|  |  |  | day 1 | 61 ± 18* | 70 ± 5* |
|  |  |  | day 3 | 73 ± 14 | 79 ± 5 |
|  | INTEM | 84 ± 1 | 4h | 71 ± 8* | 70 ± 10* |
|  |  |  | day 1 | 69 ± 20* | 75 ± 8* |
|  |  |  | day 3 | 80 ± 8 | 80 ± 6 |
|  | FIBTEM | 76 ± 2 | 4h | 11 ± 27 | 11 ± 24* |
|  |  |  | day 1 | 37 ± 40 | 56 ± 33 |
|  |  |  | day 3 | 61 ± 35 | 58 ± 39 |
| A10, mm | EXTEM | 69 ± 2 | 4h | 45 ± 8* | 44 ± 12* |
|  |  |  | day 1 | 43 ± 19* | 52 ± 9* |
|  |  |  | day 3 | 57 ± 22 | 66 ± 13 |
|  | INTEM | 71 ± 2 | 4h | 54 ± 9* | 50 ± 11* |
|  |  |  | day 1 | 54 ± 19* | 65 ± 5 |
|  |  |  | day 3 | 73 ± 9 | 69 ± 12 |
|  | FIBTEM | 12 ± 1 | 4h | 11 ± 3 | 11 ± 2 |
|  |  |  | day 1 | 16 ± 9 | 24 ± 5* |
|  |  |  | day 3 | 21 ± 11 | 24 ± 8* |
| MCF, mm | EXTEM | 73 ± 1 | 4h | 51 ± 8* | 47 ± 13* |
|  |  |  | day 1 | 51 ± 18* | 62 ± 5* |
|  |  |  | day 3 | 63 ± 21 | 70 ± 11 |
|  | INTEM | 75 ± 1 | 4h | 61 ± 6* | 58 ± 9* |
|  |  |  | day 1 | 59 ± 19 | 74 ± 2 |
|  |  |  | day 3 | 76 ± 8 | 74 ± 9 |
|  | FIBTEM | 13 ± 1 | 4h | 12 ± 3 | 12 ± 2 |
|  |  |  | day 1 | 18 ± 9 | 27 ± 3* |
|  |  |  | day 3 | 23 ± 10 | 26 ± 8* |
| ML, % | EXTEM | 4 ± 2 | 4h | 6 ± 5 | 5 ± 8 |
|  |  |  | day 1 | 3 ± 5 | 2 ± 2 |
|  |  |  | day 3 | 2 ± 3 | 6 ± 2 |
|  | INTEM | 4 ± 1 | 4h | 2 ± 3 | 1 ± 1* |
|  |  |  | day 1 | 2 ± 3 | 1 ± 1* |
|  |  |  | day 3 | 3 ± 2 | 2 ± 2 |
|  | FIBTEM | 0 ± 0 | 4h | 1 ± 2 | 2 ± 2 |
|  |  |  | day 1 | 1 ± 1 | 2 ± 3 |
|  |  |  | day 3 | 0 ± 1 | 0 ± 0 |

Data represent mean ± standard deviation. CT = clot time; α = alpha angle; A10 = clot amplitude 10 min after clot initiation; MCF = maximum clot formation; ML = maximum lysis. *p < 0.05 compared to Baseline. n=6 per group; Baseline values were obtained from tail vein of 8 healthy anaesthetized rats.
