# Supplementary material for: Comparison of intra-articular administration of adenosine, lidocaine and magnesium solution and tranexamic acid for alleviating postoperative inflammation and joint fibrosis in an experimental model of knee arthroplasty
Source: J Orthop Surg Res. 2021 Dec 20;16:726. doi: 10.1186/s13018-021-02871-y (PMC8686251; doi:10.1186/s13018-021-02871-y)
Supplement: Supplementary file 2 — Additional file 2: Table S2. Postoperative clinical, haematology and systemic inflammatory parameters. [file 13018_2021_2871_MOESM2_ESM.docx]

**Table S2.** Postoperative haematology and systemic inflammatory parameters

| **Indices** | Baseline | Time | TXA | ALM |
| --- | --- | --- | --- | --- |
| WBC, x 10^9^cells/L | 11.0 ± 4.1 | 4h  day 1  day 3  day 5  day 28 | 12.2 ± 2.6  12.8 ± 2.6  12.5 ± 2.8  11.6 ± 4.1  12.1 ± 4.4 | 13.6 ± 3.6  14.5 ± 2.4  13.5 ± 4.5  13.0 ± 4.5  10.2 ± 5.0 |
| Lymphocytes,  x 10^9^ cells/L | 7.5 ± 2.0 | 4h  day 1  day 3  day 5  day 28 | 4.2 ± 2.1^  4.9 ± 1.4^  6.7 ± 1.9  8.3 ± 2.5  7.8 ± 2.7 | 4.4 ± 2.0^  5.4 ± 1.7  7.6 ± 2.5  8.5 ± 2.8  6.9 ± 3.2 |
| Monocytes,  x 10^9^cells/L | 0.5 ± 0.7 | 4h  day 1  day 3  day 5  day 28 | 0.9 ± 0.6  1.3 ± 0.4^  0.7 ± 0.9  0.8 ± 0.7  0.8 ± 0.8 | 0.6 ± 0.7  0.8 ± 0.3  1.1 ± 1.0  1.1 ± 0.8  0.6 ± 0.6 |
| Granulocytes  x 10^9^cells/L | 3.0 ± 2.0 | 4h  day 1  day 3  day 5  day 28 | 7.1 ± 1.7^  6.7 ± 1.5^  5.1 ± 1.7  2.5 ± 1.1  3.5 ± 1.5 | 8.6 ± 2.1^  8.3 ± 1.2^  4.9 ± 1.6  3.3 ± 1.2  2.7 ± 1.4 |
| Platelets, x 10^9^/L | 193 ± 212 | 4h  day 1  day 3  day 5  day 28 | 196 ± 203  187 ± 160  136 ± 159  309 ± 189  207 ± 179 | 206 ± 208  210 ± 166  179 ± 138  165 ± 163*  272 ± 178 |
| Hematocrit, % | 41.6 ± 2.3 | 4h  day 1  day 3  day 5  day 28 | 37.9 ± 4.3  38.3 ± 1.7  37.8 ± 1.5  38.4 ± 3.1  40.2 ± 2.6 | 37.8 ± 2.2  36.0 ± 5.1^  38.5 ± 2.0  38.7 ± 2.5  41.0 ± 1.4 |
| Hgb, g/dL | 13.6 ± 0.5 | 4h  day 1  day 3  day 5  day 28 | 13.0 ± 1.5  12.5 ± 0.7^  11.6 ± 0.8^  12.2 ± 1.0^  14.0 ± 1.3 | 13.1 ± 0.9  12.0 ± 1.9^  11.6 ± 0.7^  12.4 ± 0.8^  14.0 ± 0.5 |
| MCP-1, pg/ml | 186.9 ± 53.6 | 4h  day 1  day 3  day 5  day 28 | 234.3 ± 63.3  272.4 ± 82.0^  318.1 ± 158.8^  212.4 ± 65.7  240.1 ± 156.7 | 208.8 ± 29.5  260.5 ± 51.5^  272.1 ± 49.3^  215.7 ± 66.6  210.6 ± 43.2 |
| TNF-α, pg/ml | n.d. | 4h  day 1  day 3  day 5  day 28 | n.d.  n.d.  n.d.  n.d.  n.d. | n.d.  n.d.  n.d.  n.d.  n.d. |
| IL-1β, pg/ml | 5.7 ± 3.8 | 4h  day 1  day 3  day 5  day 28 | 3.5 ± 1.0  9.0 ± 7.6  4.1 ± 1.8  4.4 ± 2.2  12.4 ± 8.0^ | 9.7 ±12.9  10.6 ± 8.8  5.7 ± 2.3  5.0 ± 5.3  7.4 ± 4.2 |
| IFN-γ, pg/ml | n.d.^#^ | 4h  day 1  day 3  day 5  day 28 | 31.1 ± 18.1  23.0 ± 11.9  20.6 ± 13.0  21.9 ± 9.7^  n.d. | 32.7 ± 21.3^  n.d.  n.d.  16.5 ± 3.0  n.d. |
| IL-4, pg/ml | 13.1 ± 8.1 | 4h  day 1  day 3  day 5  day 28 | 11.0 ± 7.5  12.7 ± 9.2  10.7 ± 4.4  14.9 ± 12.5  15.8 ± 10.4 | 19.5 ± 16.7  n.d.  6.7 ± 3.8  8.7 ± 10.5  16.5 ± 16.5 |
| sICAM, ng/ml | 8.1 ± 1.5 | 4h  day 1 | 12.4 ± 2.7^  11.0 ± 1.2^ | 10.5 ± 6.1  9.6 ± 4.9 |
| sE-selectin, ng/ml | 9.6 ± 2.3 | 4h  day 1 | 19.9 ± 7.3^  18.8 ± 6.6^ | 20.5 ± 4.2^  20.8 ± 4.3^ |
| Adiponectin, ng/ml | 42.1 ± 11.9 | 4h  day 1 | 100.8 ± 22.3^  78.3 ± 17.8^ | 86.8 ± 19.2^  85.5 ± 33.3^ |
| vWF, ng/ml | 17.3 ± 15.2 | 4h  day 1 | 115.3 ± 34.0^  104.0 ± 27.2^ | 113.4 ± 9.1^  105.4 ± 37.1^ |

Values represent mean ± standard deviation. Baseline values were obtained from 8 healthy male Sprague-Dawley rats. WBC, white blood cell count; Hgb, haemoglobin; MCP-1, monocyte chemoattractant protein 1; TNF-α, tumor necrosis factor alpha; IL, interleukin; sICAM, soluble intercellular adhesion molecule; E-selectin, endothelial-leukocyte adhesion molecule; vWF, von Willebrand factor. ^ p < 0.05 compared to baseline; * p < 0.05 compared to TXA.
