# Supplementary material for: Comparison of intra-articular administration of adenosine, lidocaine and magnesium solution and tranexamic acid for alleviating postoperative inflammation and joint fibrosis in an experimental model of knee arthroplasty
Source: J Orthop Surg Res. 2021 Dec 20;16:726. doi: 10.1186/s13018-021-02871-y (PMC8686251; doi:10.1186/s13018-021-02871-y)
Supplement: Supplementary file 3 — Additional file 3: Table S3. Inflammatory cytokines in joint capsular tissue. [file 13018_2021_2871_MOESM3_ESM.docx]

**Table S3.** Inflammatory chemokines and cytokines in joint capsule tissue in native knees, and implanted knees day 28 post-surgery.

|  | Native Knee | Implanted Knee | |
| --- | --- | --- | --- |
|  |  | TXA | ALM |
| MCP-1 | n.d. | n.d. | n.d. |
| MIP-1α | n.d. | n.d. | n.d. |
| IL-1α | n.d. | n.d. | n.d. |
| IL-1β | 19.2 ± 16.0 | 30.8 ± 52.0 | 35.7 ± 34.3 |
| TNF-α | n.d. | n.d. | n.d. |
| IL-6 | n.d. | n.d. | n.d. |
| IL-10 | n.d. | n.d. | n.d. |
| IL-4 | n.d. | n.d. | n.d. |
| IFN-γ | n.d. | n.d. | n.d. |
| RANTES | 23.3 ± 9.7 | 77.9 ± 42.5^ | 156.5 ± 105.8^ |
| IL-13 | n.d. | n.d. | n.d. |

Data is expressed as pg/mg tissue, and represent mean ± standard deviation. n.d., values below assay detection limit. MCP-1, monocyte chemoattractant protein 1; MIP-1α, macrophage inflammatory protein-1 alpha; TNF-α, tumor necrosis factor alpha; IL, interleukin; IFN-γ, interferon gamma; RANTES, regulated on activation, normal T cell expressed and secreted. ^ p < 0.05 compared to native knees.
